# Safety, Growth, and Development After Dapagliflozin or Saxagliptin in Children With Type 2 Diabetes (T2NOW Follow-Up)

**DOI:** 10.1210/clinem/dgae723

**Published:** 2024-10-24

**Authors:** Naim Shehadeh, Pietro Galassetti, Nayyar Iqbal, Cecilia Karlsson, John Monyak, Jennifer Ostridge, Marie Bolin, Timothy Barrett

**Affiliations:** Azrieli Faculty of Medicine, Bar-Ilan University, Safed, 1311502, Israel; Late-Stage Development, Cardiovascular, Renal, and Metabolism, BioPharmaceuticals R&D, AstraZeneca, Gaithersburg, MD 20878, USA; Late-Stage Development, Cardiovascular, Renal, and Metabolism, BioPharmaceuticals R&D, AstraZeneca, Gaithersburg, MD 20878, USA; Late-Stage Development, Cardiovascular, Renal, and Metabolism, BioPharmaceuticals R&D, AstraZeneca, Gothenburg, 43183, Sweden; Late-Stage Development, Cardiovascular, Renal, and Metabolism, BioPharmaceuticals R&D, AstraZeneca, Gaithersburg, MD 20878, USA; Late-Stage Development, Cardiovascular, Renal, and Metabolism, BioPharmaceuticals R&D, AstraZeneca, Gothenburg, 43183, Sweden; Late-Stage Development, Cardiovascular, Renal, and Metabolism, BioPharmaceuticals R&D, AstraZeneca, Gothenburg, 43183, Sweden; Department of Cancer and Genomic Sciences, College of Medicine and Health, University of Birmingham, Birmingham B15 2TT, UK

**Keywords:** SGLT2 inhibitor, DPP4 inhibitor, dapagliflozin, saxagliptin, type 2 diabetes, pediatric

## Abstract

**Context:**

The T2NOW trial of dapagliflozin or saxagliptin vs placebo in pediatric patients with type 2 diabetes (T2D) demonstrated promising efficacy data for dapagliflozin and did not raise any safety concerns over 52 weeks.

**Objective:**

This work aimed to assess long-term effects of prior dapagliflozin/saxagliptin administration on safety, growth, and development.

**Methods:**

A multicenter, randomized, double-blind phase 3 trial (T2NOW) was conducted among 210 children with T2D aged 10 to 17 years, followed for up to 1 year after treatment. Participants were previously treated with once-daily dapagliflozin (5, 10 mg), saxagliptin (2.5, 5 mg), or placebo as an add-on to diet, exercise, metformin, and/or insulin for 52 weeks, plus a 52-week nontreatment follow-up period. Main outcome measures included change in height, weight, body mass index (BMI), Tanner staging, growth and maturation markers, bone biomarkers, and adverse events (AEs) from baseline to week 104.

**Results:**

As expected in a pediatric population, mean height and weight slightly increased from baseline to week 104. BMI remained generally stable; changes were similar across treatment groups. Sexual maturation progressed normally to week 104, with similar shifts between Tanner stages and changes in growth and maturation markers and bone biomarkers across groups. The proportion of patients reporting 1 or more AEs during the nontreatment follow-up period was similar across groups previously treated with dapagliflozin (18.5%) or saxagliptin (15.9%) compared to placebo (21.1%). No deaths occurred.

**Conclusion:**

Prior treatment with dapagliflozin or saxagliptin for 52 weeks did not raise any safety concerns relating to height, weight, BMI, Tanner staging, growth and maturation markers, bone biomarkers, or AEs for up to 52 weeks following treatment discontinuation in pediatric patients with T2D.

The global incidence and prevalence of type 2 diabetes (T2D) are increasing among children and adolescents, with approximately 41 600 new cases of T2D diagnosed in this pediatric population worldwide in 2021 (1, 2). When T2D is diagnosed in youth, the disease is more progressive and aggressive than adult-onset T2D, with an earlier onset of complications and a shorter time before resistance develops to standard treatment options (2-6).

Despite this global increase in incidence of T2D in children and adolescents, the number of approved treatments for this population is limited compared with those available for adults (3). Management of T2D in children and adolescents typically involves healthy lifestyle changes and pharmaceutical intervention, with metformin and add-on insulin currently the first-line therapy for most youth with T2D (3, 6). The US Food and Drug Administration and European Medicines Agency have recently approved a new drug class, injectable glucagon-like peptide-1 receptor agonist (GLP-1 RA) therapies (eg, once-daily liraglutide (7), once-weekly exenatide (8), and once-weekly dulaglutide) (9), for use in pediatric T2D for treatment alone or in combination with metformin and/or insulin (3, 6). However, these medications have been reported to increase the frequency of some adverse reactions (eg, gastrointestinal intolerance, including abdominal discomfort, nausea, vomiting, and diarrhea) (6, 10), and adherence challenges of injectable therapies are further exacerbated in young patients (as seen in adherence to insulin therapy) (6, 11), highlighting the urgent need for novel oral treatment options with fewer adverse effects for pediatric patients with T2D.

Dapagliflozin is the first oral treatment since metformin to be approved for use in children with T2D aged 10 and older and was approved by the European Medicines Agency for this indication in 2022 (12). It is a highly potent, reversible, and selective sodium-glucose cotransporter-2 (SGLT-2) inhibitor that works by inhibiting the action of this main transporter (responsible for glucose absorption in the proximal tubule) to increase the amount of glucose excreted from the body (13). Saxagliptin is a dipeptidyl peptidase-4 (DPP-4) inhibitor, another oral therapy that was first approved by the Food and Drug Administration for treatment of T2D in adults in 2006 (14, 15). DPP-4 inhibitors selectively inhibit the DPP-4 enzyme that normally degrades 2 major incretin hormones, glucagon-like peptide-1 and glucose-dependent insulinotropic polypeptide (14, 15).

T2NOW was a pivotal phase 3 trial which assessed the efficacy of dapagliflozin and saxagliptin vs placebo in patients aged 10 to 17 years with T2D (16). Patients treated with dapagliflozin achieved decreases in glycated hemoglobin A_1c_ (HbA_1c_) and fasting plasma glucose (FPG) from as early as 6 weeks of therapy, which were sustained over 52 weeks. In the primary analysis at week 26, dapagliflozin demonstrated a significant reduction in HbA_1c_ levels, with an adjusted mean change of −0.62% (95% CI, −1.05 to −0.19), compared to an increase of +0.41% (95% CI, −0.01 to 0.84) in the placebo group. This resulted in a notable placebo-subtracted difference of −1.03% (95% CI, −1.57 to −0.49; *P* < .001), demonstrating the effect of dapagliflozin on glycemic control. Patients treated with saxagliptin did not achieve statistical significance in the primary analysis at week 26.

In addition to studying the efficacy of these novel oral treatment options, it is also important to assess their long-term safety for use in children and adolescents. Safety outcomes assessed over 52 weeks in the T2NOW trial indicated that dapagliflozin and saxagliptin were both well tolerated in a pediatric population over this period with no significant safety concerns (16). The proportion of participants with one or more adverse events (AEs) was similar in the dapagliflozin and also the saxagliptin treatment groups when each was compared with placebo, although nonserious headaches were more common in participants treated with dapagliflozin compared to placebo. The proportion of participants reporting hypoglycemia was similar across groups; no deaths occurred. There was no notable effect of dapagliflozin or saxagliptin observed on measures of growth and maturation at week 52. However, the long-term effects of active treatment with dapagliflozin or saxagliptin on children and adolescents have not previously been investigated.

Here we report results from the 52-week nontreatment follow-up period of T2NOW, describing measures of growth and maturity, Tanner staging, markers of bone health, and safety following withdrawal of dapagliflozin or saxagliptin in children and adolescents with T2D.

## Materials and Methods

### Study Design and Participants

**Figure 1.**
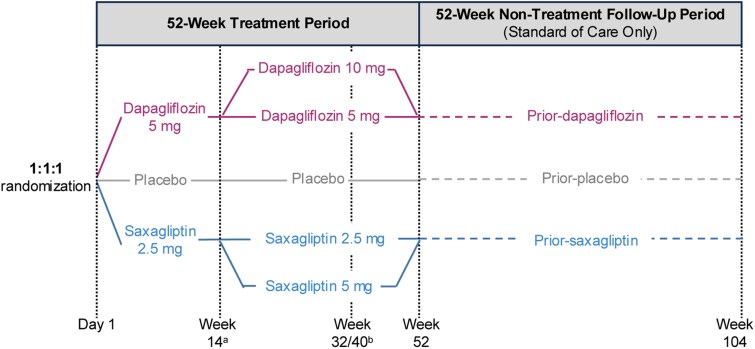
Study design. ^a^Patients who were receiving active drug and had not achieved the glycemic target of a glycated hemoglobin A_1c_ (HbA_1c_) less than 7% at week 12 were randomly assigned at week 14 to either continue their current dose or increase the dose (dapagliflozin 10 mg or saxagliptin 5 mg); patients on placebo continued on placebo; ^b^a third randomization occurred at week 32/40 with patients receiving background treatment with metformin only who had an HbA_1c_ of less than 7.5% at week 26/32. Patients who were receiving active drug were randomly assigned to either continue background metformin or discontinue and increase the dose of active drug if they were on the low dose. Patients who were receiving placebo were randomly assigned to either discontinue background metformin and switch to active treatment (dapagliflozin 10 mg or saxagliptin 5 mg) or continue background metformin and remain on placebo.

The study design for T2NOW (NCT03199053), a multicenter, randomized, double-blind, phase 3 trial comparing dapagliflozin and saxagliptin treatment vs placebo, has been reported previously (Fig. 1) (16).

Eligible participants were pediatric patients with T2D aged 10 to <18 years, with HbA_1c_ 6.5% to 10.5% on diet and exercise and a stable dose of metformin (≥1000 mg), insulin, or metformin (≥1000 mg) plus insulin at enrollment. Parents or guardians provided written informed consent, and patients provided written assent as appropriate. The trial was approved by institutional review boards and independent ethics committees associated with each center. Full inclusion and exclusion criteria have been published previously (16).

The study consisted of a 52-week treatment period, results of which have been published previously (16), followed by a 52-week nontreatment follow-up period. During the treatment period patients were initially randomly assigned 1:1:1 to oral once-daily dapagliflozin (5 mg), saxagliptin (2.5 mg), or placebo, in addition to background treatment (metformin only, insulin only, or metformin plus insulin). There were two further randomizations during the treatment period. Patients who were receiving the active drug and had not achieved the glycemic target of HbA_1c_ of less than 7% at week 12 were randomly assigned to either continue their current dose or increase the dose (dapagliflozin 10 mg or saxagliptin 5 mg) at week 14; patients on placebo continued on placebo. At week 32 or week 40, patients receiving background treatment with metformin only with HbA_1c_ of less than 7.5% at week 26 or week 32 were further randomly assigned, provided they had not initiated rescue glycemic control therapy. Patients who were receiving active drug either continued or discontinued background metformin; patients who discontinued metformin and were on low-dose active drug had their dose increased (dapagliflozin 10 mg or saxagliptin 5 mg). Patients who were receiving placebo either discontinued background treatment and switched to active treatment with either dapagliflozin 10 mg or saxagliptin 5 mg, or continued background treatment with metformin and remained on placebo.

Following the treatment period, patients continued into a 52-week nontreatment follow-up period. The nontreatment follow-up period started the day after the end of the treatment period and ended on the week 104 visit date or the date of the last assessment if the patient was lost to follow-up, whichever was earlier. During the follow-up period, patients stopped receiving the study drug and continued standard of care treatment, consisting of any approved medication, at the discretion of the treating physician.

It should be noted that, in a small number of patients, the week 52 visit was performed up to several weeks past the calendar 52-week time point due to unforeseen circumstances (including the COVID pandemic). In these cases, the week 104 visit was still maintained at the correct calendar date, rendering the interval between the week 52 and the week 104 visits less than 52 weeks.

### Outcomes

Height, weight, body mass index (BMI), Tanner staging, puberty status, growth and maturation markers (25-hydroxyvitamin D, calcitonin, insulin-like growth factor [IGF]-1, IGF binding protein [IGFBP]-3, follicle stimulating hormone [FSH], luteinizing hormone, estradiol, testosterone, free thyroxine [T4]), and bone biomarkers (alkaline phosphatase [ALP], osteocalcin, parathyroid hormone [PTH], C-terminal telopeptide [CTX]) were assessed during the initial 52-week treatment period and at the week 104 visit. Normalized values for BMI (*z* scores) were produced by adjusting for age and sex using US Centers for Disease Control and Prevention growth charts.

Safety was assessed throughout the 52-week nontreatment follow-up period, from the day after the treatment period ended to the week 104 visit, or the date of the last assessment if the patient was lost to follow-up; AEs that occurred during the initial 52-week treatment period are not reported again here. AEs were collected quarterly via phone assessment during the 52-week follow-up period. All events were classified according to the Medical Dictionary for Regulatory Activities (MedDRA) version 25.1. AEs of special interest comprised a predefined list of preferred terms (16). Hypoglycemia was defined according to American Diabetes Association and International Society for Pediatric and Adolescent Diabetes Classification (17, 18).

Data are presented for all patients who received 1 or more treatment doses during the treatment period of T2NOW. Observed data are reported for all outcomes except Tanner staging, growth and maturation markers, and bone biomarkers, for which last observation carried forward (LOCF) imputation was used if the relevant postbaseline visit was missing. Data are presented for patients grouped by the first actual treatment received during the treatment phase, that is, the study drug assigned during the first randomization: dapagliflozin (5 mg; “prior dapagliflozin” group), saxagliptin (2.5 mg; “prior saxagliptin” group), or placebo (“prior placebo” group). The word “prior” prefixes each of the treatments since this analysis is focused on the nontreatment follow-up. However, in cases where baseline or time points up to week 52 are displayed, this of course reflects actual rather than prior usage of the treatments. The number of re-randomly assigned patients reassigned from the placebo group to active study drug at week 32 or week 40 was small (n = 3 placebo to dapagliflozin, n = 3 placebo to saxagliptin), and analyses of patients grouped by last actual treatment showed very little difference compared to analyses by first actual treatment.

## Results

### Patient Disposition and Baseline Characteristics

A total of 245 patients with T2D were randomly assigned to the T2NOW study. Baseline demographic and characteristics for patients have been published previously and were similar between treatment groups and the placebo group and representative of a young population with T2D (16). The average age was 14.5 years and 59.6% of patients were female. Around half of patients (50.6%) were White, 26.5% Asian, 12.2% American Indian/Alaska Native, and 5.7% Black or African American; the remaining patients (4.9%) comprised other populations such as Native Hawaiian/other Pacific Islander/Other. The majority of patients were on metformin-only background medication (51.4%), followed by insulin plus metformin (36.3%) and insulin alone (12.2%).

**Figure 2.**
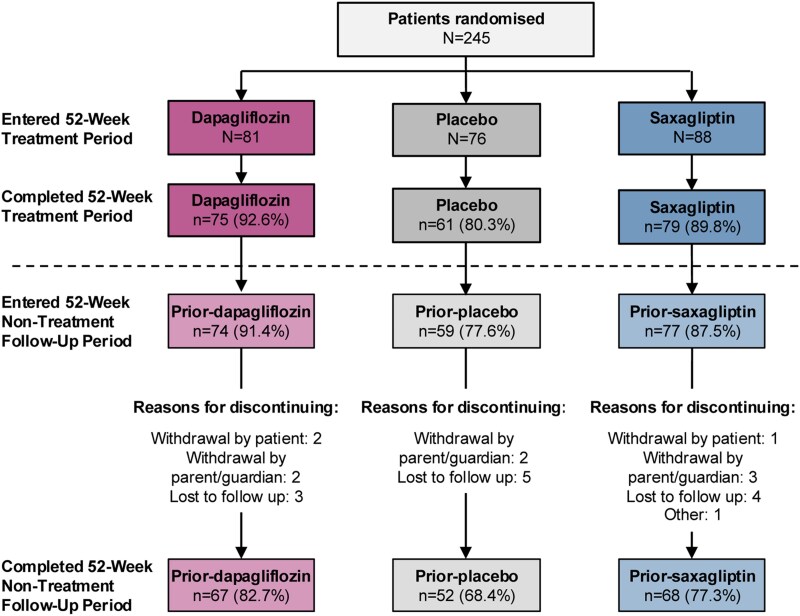
Patient disposition and discontinuations. Eight patients in the placebo group were re-randomly assigned at week 32 or week 40: Two patients remained in the placebo group, 3 patients were reassigned to the dapagliflozin group, and 3 patients to the saxagliptin group.

Of the 245 patients initially randomly assigned to the 52-week treatment period, 210 (85.7%) entered the 52-week nontreatment follow-up period and 187 (76.3% of the original patients) completed the week 104 visit (Fig. 2). A similar proportion of randomly assigned patients from the dapagliflozin (91.4%) and saxagliptin (87.5%) groups entered the follow-up period, whereas a lower proportion of patients entered the follow-up period from the placebo group (77.6%). During the 52-week nontreatment follow-up period, 23 patients (9.4%) withdrew from the study (n = 7 for prior dapagliflozin, n = 9 for prior saxagliptin, n = 7 for prior placebo). The most frequent reason for withdrawal was loss to follow-up (n = 12). A total of 187 (76.3%) patients (n = 67 for prior dapagliflozin, n = 68 for prior saxagliptin, n = 52 for prior placebo) completed the week 104 visit.

During the follow-up period patients received standard of care, at the discretion of the treating physician. Concomitant medications were reported for 7 (8.6%), 12 (13.6%), and 14 (18.4%) patients in the prior dapagliflozin, prior saxagliptin, and prior placebo groups, respectively. Apart from metformin and insulin analogues, the most common concomitant medications were paracetamol (6 [2.4%] patients), COVID-19 vaccines (4 [1.6%] patients), and nonsteroidal anti-inflammatory medicines (4 [1.6%] patients).

Standard of care changed during the study, from diet and exercise with metformin, insulin, or metformin plus insulin to also include a number of newly approved treatments including liraglutide, dulaglutide, exenatide, empagliflozin and, in some markets (including the European Union), dapagliflozin. Of these, liraglutide was reported for 1 patient in each of the prior dapagliflozin, prior saxagliptin, and prior placebo groups, and semaglutide was reported for 1 patient in the prior placebo group.

### Physical Findings: Height, Weight, and Body Mass Index

**Figure 3.**
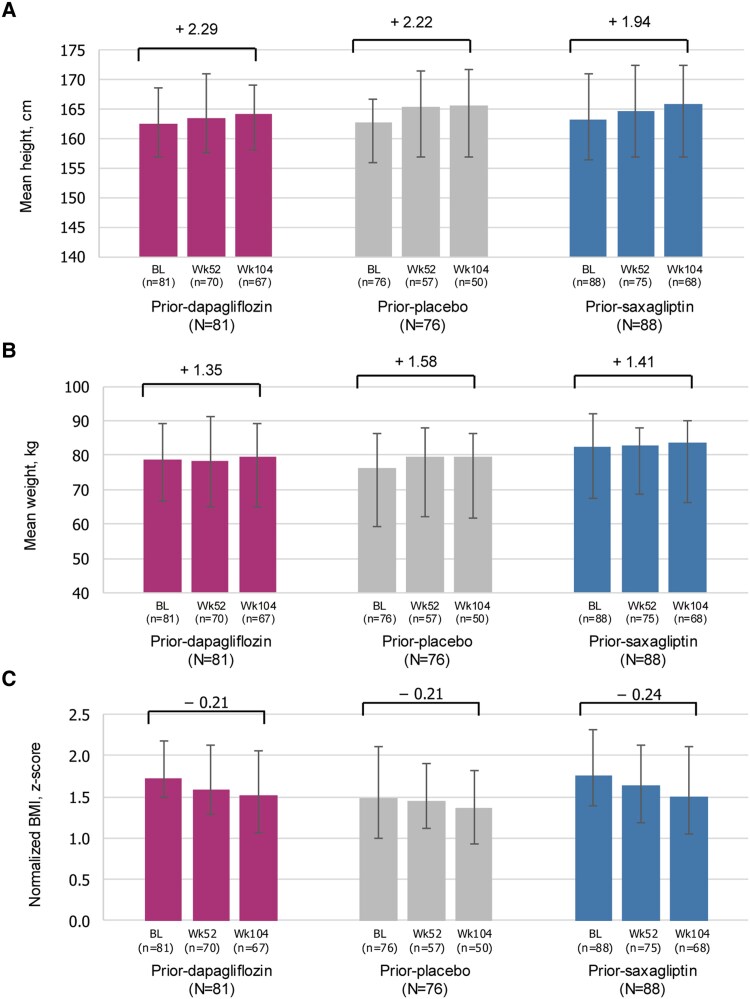
Growth assessments over time. Data presented as mean height, mean weight, or normalized BMI; error bars represent IQR. Abbreviations: BL, baseline; BMI, body mass index; IQR, interquartile range; Wk, week.

As expected in a pediatric population, small increases in mean height and mean weight were observed over the treatment period and the 52-week nontreatment follow-up period. The average change in height from baseline was similar at week 104 between each of the prior dapagliflozin (2.29 cm) and prior saxagliptin (1.94 cm) groups compared to prior placebo (2.22 cm; Fig. 3A). The mean gain in weight between baseline and the week 104 visit was also similar in each of the prior dapagliflozin (1.35 kg) and prior saxagliptin (1.41 kg) groups compared to the prior placebo group (1.58 kg; Fig. 3B). Changes in mean BMI (*z* score) from baseline to week 104 were small and similar between each of the prior dapagliflozin (−0.21) and prior saxagliptin (−0.24) groups compared to the prior placebo group (−0.21; Fig. 3C).

### Tanner Staging and Puberty Status

Tanner staging (sexual maturity rating) was used as an objective classification system to track the development of secondary sex characteristics and puberty in the patient population, during treatment with the study drug and then over the 52 weeks following its withdrawal. As expected in this young population, there was a shift in Tanner stages throughout the study (Table 1). Similar patterns were seen among patients who had previously been treated with either dapagliflozin or saxagliptin and those who had received placebo. A normal progression of sexual maturation was observed both over the treatment period and the 52-week nontreatment follow-up period.

**Table 1.** Tanner stages over time

		No. (%) of patients						
		Tanner stage at wk 104						
Treatment group	Baseline Tanner stage	Stage 1	Stage 2	Stage 3	Stage 4	Stage 5	Not reported	Total
**Prior dapagliflozin (N = 81)**	Stage 1	0	1 (1.2)	0	0	0	0	1 (1.2)
	Stage 2	0	0	6 (7.4)	3 (3.7)	1 (1.2)	1 (1.2)	11 (13.6)
	Stage 3	0	0	2 (2.5)	8 (9.9)	1 (1.2)	0	11 (13.6)
	Stage 4	0	0	0	7 (8.6)	19 (23.5)	3 (3.7)	29 (35.8)
	Stage 5	0	0	0	0	26 (32.1)	2 (2.5)	28 (34.6)
	Not reported	0	0	0	0	1 (1.2)	0	1 (1.2)
	Total	0	1 (1.2)	8 (9.9)	18 (22.2)	48 (59.3)	6 (7.4)	81 (100)
**Prior placebo (N = 76)**	Stage 1	0	0	1 (1.3)	0	0	0	1 (1.3)
	Stage 2	0	2 (2.6)	3 (3.9)	2 (2.6)	0	0	7 (9.2)
	Stage 3	0	0	4 (5.3)	3 (3.9)	3 (3.9)	0	10 (13.2)
	Stage 4	0	0	0	5 (6.6)	10 (13.2)	0	15 (19.7)
	Stage 5	0	0	0	1 (1.3)	39 (51.3)	2 (2.6)	42 (55.3)
	Not reported	0	0	0	0	0	1 (1.3)	1 (1.3)
	Total	0	2 (2.6)	8 (10.5)	11 (14.5)	52 (68.4)	3 (3.9)	76 (100)
**Prior-saxagliptin (N = 88)**	Stage 1	1 (1.1)	1 (1.1)	0	1 (1.1)	0	0	3 (3.4)
	Stage 2	0	0	2 (2.3)	3 (3.4)	0	0	5 (5.7)
	Stage 3	0	0	5 (5.7)	5 (5.7)	2 (2.3)	0	12 (13.6)
	Stage 4	0	0	1 (1.1)	8 (9.1)	14 (15.9)	1 (1.1)	24 (27.3)
	Stage 5	0	0	0	0	44 (50.0)	0	44 (50.0)
	Not reported	0	0	0	0	0	0	0
	Total	1 (1.1)	1 (1.1)	8 (9.1)	17 (19.3)	60 (68.2)	1 (1.1)	88 (100)

Missing postbaseline data are imputed using last observation carried forward.

### Growth and Maturation Markers and Bone Biomarkers

The pattern of changes in growth and maturation markers and biomarkers of bone health from baseline over the treatment period and the 52-week nontreatment follow-up period was similar between each of the prior dapagliflozin and prior saxagliptin groups compared to the prior placebo group (Table 2).

**Table 2.** Growth and maturation markers and bone biomarkers over time

Growth and maturation markers, mean (SD)				
Visit	Marker	Prior dapagliflozin (N = 81)	Prior placebo (N = 76)	Prior saxagliptin (N = 88)
**Baseline**	25-Hydroxyvitamin D (nmol/L)	48.8 (17.9)	47.1 (17.0)	46.9 (18.6)
	Calcitonin, ng/L	2.3 (1.1)	2.2 (0.9)	2.2 (1.1)
	Estradiol, pmol/L	238.3 (208.5)	205.3 (196.3)	234.0 (198.3)
	FSH, IU/L	5.6 (5.4)	5.8 (6.4)	6.6 (9.3)
	IGFBP-3, mg/L	6.5 (1.6)	6.7 (1.5)	6.3 (1.4)
	IGF-1, µg/L	277.4 (102.2)	283.9 (105.2)	282.1 (104.1)
	LH, IU/L	7.5 (11.2)	7.1 (12.9)	6.6 (10.2)
	Testosterone, nmol/L	5.3 (6.8)	5.2 (6.8)	6.0 (7.4)
	Thyrotropin, mIU/L	2.2 (1.2)	2.1 (1.0)	2.3 (1.4)
	T4, pmol/L	12.6 (2.1)	12.1 (2.4)	12.2 (2.1)
**Week 52**	25-hydroxyvitamin D (nmol/L)	48.5 (17.7)	49.1 (18.1)	47.1 (19.0)
	Calcitonin, ng/L	2.2 (1.1)	2.4 (1.9)	2.1 (0.4)
	Estradiol, pmol/L	215.3 (190.6)	206.5 (188.2)	257.7 (250.3)
	FSH, IU/L	6.0 (7.0)	5.3 (2.9)	7.5 (10.7)
	IGFBP-3, mg/L	6.3 (1.6)	6.8 (1.7)	6.3 (1.6)
	IGF-1, µg/L	256.2 (95.0)	253.3 (84.9)	257.4 (93.2)
	LH, IU/L	6.2 (6.3)	5.8 (4.9)	8.2 (9.3)
	Testosterone, nmol/L	6.0 (7.9)	6.5 (7.5)	5.6 (6.8)
	Thyrotropin, mIU/L	2.0 (1.2)	2.6 (3.9)	2.6 (3.2)
	T4, pmol/L	13.2 (4.7)	12.2 (1.8)	12.4 (1.8)
**Week 104**	25-Hydroxyvitamin D (nmol/L)	45.7 (22.8)	45.5 (15.3)	45.3 (18.7)
	Calcitonin, ng/L	2.3 (1.3)	2.3 (0.9)	2.1 (0.3)
	Estradiol, pmol/L	216.0 (253.1)	214.3 (177.7)	226.0 (207.7)
	FSH, IU/L	5.5 (4.1)	5.1 (3.1)	6.3 (7.5)
	IGFBP-3, mg/L	6.4 (2.0)	6.7 (1.5)	6.4 (1.6)
	IGF-1, µg/L	228.5 (90.1)	245.7 (79.3)	239.0 (85.6)
	LH, IU/L	6.5 (7.1)	6.5 (6.5)	7.9 (12.8)
	Testosterone, nmol/L	6.1 (7.9)	6.2 (7.4)	5.8 (7.5)
	Thyrotropin, mIU/L	2.5 (3.7)	2.0 (1.2)	3.0 (4.0)
	T4, pmol/L	12.9 (2.5)	12.5 (2.5)	12.5 (1.9)

Bone biomarkers, mean (SD)				
Visit	Biomarker	Prior dapagliflozin (N = 81)	Prior placebo (N = 76)	Prior-saxagliptin (N = 88)
**Baseline**	ALP, µ/L	35.1 (31.2)	32.6 (23.4)	31.9 (22.7)
	Osteocalcin, µg/L	43.2 (32.1)	43.0 (30.1)	40.3 (29.3)
	PTH, pmol/L	4.0 (1.8)	3.6 (1.7)	3.9 (1.7)
	CTX, µg/L	8.1 (3.7)	8.2 (4.0)	8.8 (4.8)
**Week 52**	ALP, µg/L	30.6 (25.1)	27.8 (19.5)	23.9 (14.9)
	Osteocalcin (µg/L)	39.5 (30.7)	34.6 (21.0)	32.0 (23.9)
	PTH, pmol/L	4.0 (2.0)	3.9 (1.6)	3.7 (1.6)
	CTX, µg/L	7.6 (3.9)	7.7 (5.7)	6.9 (4.0)
**Week 104**	ALP, µg/L	22.6 (16.7)	22.5 (17.0)	20.3 (11.2)
	Osteocalcin, µg/L	28.2 (20.8)	29.8 (18.3)	26.4 (14.4)
	PTH, pmol/L	4.4 (2.4)	3.9 (1.8)	3.8 (1.9)
	CTX, µg/L	6.2 (3.4)	6.4 (3.6)	6.2 (3.6)

Missing postbaseline data are imputed using LOCF.

Abbreviations: ALP, alkaline phosphatase; CTX, C-terminal telopeptide; FSH, follicle-stimulating hormone; IGF-1, insulin-like growth factor-1; IGFBP-3, IGF binding protein-3; LH, luteinizing hormone; LOCF, last observation carried forward; PTH, parathyroid hormone; T4, free thyroxine.

### Safety

Safety data for the initial 52-week treatment period of T2NOW have been reported previously and are not presented again here (16).

Following study treatment discontinuation, during the nontreatment phase, 18.4% of patients reported at least 1 AE, with a similar number of patients in each of the prior dapagliflozin (15 [18.5%]) and prior saxagliptin (14 [15.9%]) groups compared to the prior placebo group (16 [21.1%]; Table 3). The most commonly reported AEs during the nontreatment follow-up period were in the system organ class of infections and infestations, reported in 8 (9.9%), 6 (6.8%), and 8 (10.5%) patients in the prior dapagliflozin, prior saxagliptin, and prior placebo groups, respectively. The most reported AEs were upper respiratory tract infection (1 prior dapagliflozin patient, 1 prior saxagliptin patient, and 3 prior placebo patients), COVID-19 (1 prior dapagliflozin patient, 2 prior saxagliptin patients, and 2 prior placebo patients), hyperglycemia (2 prior dapagliflozin patients, 1 prior saxagliptin patient, and 1 prior placebo patient), and arthralgia (2 prior dapagliflozin patients, 0 prior saxagliptin patients, and 1 prior placebo patient).

**Table 3.** Safety data

	Prior dapagliflozin (N = 81)		Prior placebo (N = 76)		Prior saxagliptin (N = 88)	
	No. (%) of patients*^a^*	No. of events	No. (%) of patients*^a^*	No. of events	No. (%) of patients*^a^*	No. of events
AEs						
≥1 AE	15 (18.5)	37	16 (21.1)	45	14 (15.9)	22
AEs leading to discontinuation	0	0	0	0	0	0
≥1 SAE	5 (6.2)	7	5 (6.6)	11	2 (2.3)	2
≥1 AE or hypoglycemia event	15 (18.5)	37	17 (22.4)	46	14 (15.9)	22
Most common AEs*^b^*						
Upper respiratory tract infection	1 (1.2)	1	3 (3.9)	3	1 (1.1)	1
COVID-19	1 (1.2)	1	2 (2.6)	2	2 (2.3)	2
Hyperglycemia	2 (2.5)	2	1 (1.3)	1	1 (1.1)	1
Arthralgia	2 (2.5)	2	1 (1.3)	1	0	0
Hypoglycemia						
≥1 hypoglycemia event	0	0	1 (1.3)	1	0	0
≥1 hypoglycemia SAE	0	0	0	0	0	0
≥1 adjudicated DKA SAE	0	0	1 (1.3)	1	0	0
≥1 related AE	0	0	0	0	1 (1.1)	1
≥1 related SAE	0	0	0	0	0	0
≥1 AEOSI	6 (7.4)	12	9 (11.8)	18	5 (5.7)	8
Deaths	0	0	0	0	0	0

Patients with events in more than one category were counted once in each of those categories. MedDRA version 25.1.

Abbreviations: AE, adverse event; AEOSI, adverse events of special interest; DKA, diabetic ketoacidosis; MedDRA, Medical Dictionary for Regulatory Activities; SAE, serious adverse event.

^*a*^Patients with multiple events in the same category were counted only once in that category.

^*b*^Reported by more than one patient in any group.

The number of patients reporting serious AEs (SAEs) was low: 5 (6.2%) prior dapagliflozin patients reported a total of 7 SAEs, 2 (2.3%) prior saxagliptin patients reported 1 SAE each, and 5 (6.6%) prior placebo patients reported a total of 11 SAEs. None of the SAEs were considered related to the study medication. There was one hypoglycemia AE and one SAE of adjudicated diabetic ketoacidosis (DKA), both in the prior placebo group. There were no events of severe hypoglycemia or DKA reported in the prior dapagliflozin or prior saxagliptin groups. No deaths occurred during either the initial 52-week treatment period or the 52-week nontreatment follow-up period.

## Discussion

In the T2NOW trial, dapagliflozin has previously shown efficacy in improving glycemic control in children and adolescents with T2D, with statistically significant decreases in HbA_1c_ and FPG compared to placebo seen after 26 weeks of treatment (16). Here we further show that 52 weeks of treatment with dapagliflozin did not cause any safety concerns or significant longer-term effects on measures of growth and maturity, Tanner staging, and markers of bone health for up to 52 weeks following treatment discontinuation. The findings from the height, weight, BMI, Tanner staging, growth and maturation markers, bone biomarkers, and AEs did not raise any concerns in pediatric patients with T2D aged 10 to <18 years. Similarly, no safety concerns or apparent effects on measures of growth and maturity, Tanner staging, and markers of bone health were seen among patients previously treated with saxagliptin compared to those previously treated with placebo.

Unlike in adult populations, for whom body weight is generally reduced with add-on treatment with dapagliflozin or maintained with saxagliptin (13, 19), in pediatric populations an increase in body weight should be expected, in line with normal growth and development. In accordance with this, in this study we observed small increases in height and body weight from baseline, throughout the 52 weeks of active treatment and the following 52 weeks of standard-of-care treatment. Similar increases were seen across the patient groups, whether they initially received placebo or the active drug. BMI *z* scores, which provide a measure of how many standard deviations a child's BMI is above or below the average BMI for their age and sex, remained stable or slightly decreased throughout the study. This is in line with the phase 3 T2GO trial of adolescent and young adult patients with T2D treated with dapagliflozin 10 mg, where dapagliflozin treatment similarly had no notable effects on body weight or BMI *z* score compared to placebo (12).

The safety results from the 52 weeks following treatment withdrawal were consistent with those previously reported during the T2NOW treatment period and with the overall well-established safety profiles for dapagliflozin and saxagliptin in adult populations (13, 16, 20), with no new safety concerns identified.

To the best of our knowledge, these findings represent the longest-term follow-up data currently available for a treatment targeting T2D in pediatric populations, and the only safety data following discontinuation of the study drug. This is of particular importance in this population, allowing assessment of any potential effects of treatment on growth and development. The phase 3 T2GO trial assessing the efficacy of dapagliflozin 10 mg in patients with T2D aged 10 to 24 years assessed safety measures as secondary outcomes up to 52 weeks, but did not continue to assess safety after study drug discontinuation (12). The phase 3 DINAMO (Diabetes Study of Linagliptin and Empagliflozin in Children and Adolescents) trial, which assessed the efficacy of empagliflozin (an SGLT-2 inhibitor) and linagliptin (a DPP-4 inhibitor) in youth with T2D, assessed safety only until week 52 (21), again highlighting the previous lack of investigation into the longer-term safety of T2D treatment in children and adolescents after treatment withdrawal. Limitations of these findings include the small sample size of the trial, and the relatively short timeframe.

**Figure 4.**
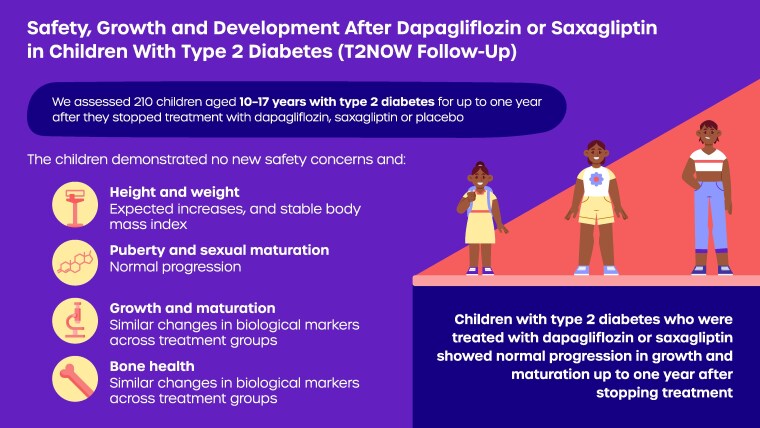
Graphical summary.

In conclusion, the 52-week nontreatment follow-up period of T2NOW provides data on the long-term safety of dapagliflozin and saxagliptin in children and adolescents aged 10 to <18 years with T2D. There were no significant effects of dapagliflozin or saxagliptin treatment on height, weight, BMI, Tanner staging, growth and maturation markers, or bone biomarkers up to 52 weeks following treatment withdrawal. No new safety concerns were identified. A graphical summary of these results is shown in Fig. 4.

## Data Availability

Some or all data sets generated during and/or analyzed during this study are not publicly available but are available from the corresponding author on reasonable request.

## Clinical Trial Information

Clinical trial registration number NCT03199053 (registered June 22, 2017).

## Abbreviations

AE: adverse event
AEOSI: adverse events of special interest
ALP: alkaline phosphatase
BMI: body mass index
CTX: C-terminal telopeptide
DKA: diabetic ketoacidosis
DPP-4: dipeptidyl peptidase-4
FPG: fasting plasma glucose
FSH: follicle-stimulating hormone
GLP-1 RA: glucagon-like peptide-1 receptor agonist
HbA_1c_: glycated hemoglobin A_1c_
IGF: insulin-like growth factor
IGFBP-3: insulin-like growth factor binding protein-3
LH: luteinizing hormone
LOCF: last observation carried forward
MedDRA: Medical Dictionary for Regulatory Activities
PTH: parathyroid hormone
SAE: serious adverse event
SGLT-2: sodium-glucose cotransporter-2
T2D: type 2 diabetes
T4: thyroxine
